# Biplanar MRI significantly improves early detection of transient global amnesia

**DOI:** 10.1007/s00415-024-12643-3

**Published:** 2024-08-31

**Authors:** Omar Alhaj Omar, Anne Mrochen, Norma J. Diel, Stefan T. Gerner, Hagen B. Huttner, Julia Heinrichs, Tobias Braun

**Affiliations:** 1 Department of Neurology, Justus-Liebig-University, Klinikstrasse 33, 35392 Giessen, Germany; 2 Center of Mind, Brain and Behavior (CMBB), Marburg, Germany; 3 Translational Neuroscience Network Giessen (TNNG), Giessen, Germany; 4 Department of Radiology, Lahn-Dill-Kliniken Wetzlar, 35578 Wetzlar, Germany; 5 Department of Neurology, Lahn-Dill-Kliniken Wetzlar, 35578 Wetzlar, Germany

## Introduction

Transient global amnesia (TGA) is defined as temporary but thorough memory loss that includes difficulty forming memories and struggling to recall events. Although this condition was observed earlier by researchers, it was officially named in 1958 by Fisher and Adams, with more details provided in their previous publication [1, 2].

In the general population, 10 out of every 100,000 individuals experience TGA annually, with the number rising to 32 cases per 100,000 for those aged 50 and above, predominantly affecting female patients [3].

The cause of TGA remains unexplained. Initially, it was thought to be linked to seizures [4]. However, this theory has not been substantiated by electroencephalographic work-up. Recent theories propose that the condition may be related to cerebral ischemia affecting specific areas of the brain, hinting at a vascular mechanism behind the condition [5, 6]. Studies have shown that the risk of having a stroke after an episode of amnesia does not seem to increase significantly compared to the risk of having a stroke after a migraine, epilepsy and transient ischemic attack, but some research suggests a slightly higher risk for stroke compared to the general population [7–9].

The causes of TGA remain unclear, resulting in a lack of objective diagnostic methods despite the German Society of Neurology’s guidelines recommending magnetic resonance imaging with axial and coronal angulation along the hippocampus’s longitudinal axis when TGA is suspected [10]. However, data supporting this approach are still inconclusive. MRI scans conducted hours after an episode have revealed areas of damage hinting at a vascular mechanism behind the condition. The clinical reference standard for diagnosing TGA is diffusion-weighted magnetic resonance imaging, which can detect small areas of restricted diffusion in one or both hippocampi. However, studies have shown that these characteristic findings are often not present in most patients when imaged early; therefore, they do not contribute to early diagnosis or decision-making. In the present study, we aimed to analyze hippocampal lesions in TGA patients, particularly during the hyperacute phase, using biplanar MRI, thereby enhancing diagnostic accuracy and management of this condition.

## Methods

This study is a retrospective analysis conducted at a single-center hospital. We analyzed routine data and MRI images of all patients with a diagnosis of TGA who underwent MRI in the period from January 2019 to March 2024. The diagnosis was made clinically. We assessed patients’ demographics, time from symptom onset to admission and time from admission to MRI. MRI images were analyzed separately by one radiologist and one neurologist, both blinded for clinical data, for occurrence of diffusion-weighted imaging (DWI) lesions in axial or coronal slices, and its location. Approval for this study was obtained from the local institutional review board (Approval Number: AZ 220/21). The board waived the need for informed consent.

## MR imaging

Magnetic resonance imaging was performed on a (1.5 T) MR system (Philips Ingenia: www.philips.de/healthcare/product/HC781341/ingenia-15t-mr-system) in all patients. A standard protocol was used in all patients, consisting of axial (field of view 180 × 231; matrix 64 × 72; slice thickness 5 mm) and coronal DWI (field of view 97 × 109; matrix 64 × 72; slice thickness 3 mm) sequences. The axial sequences covered the whole brain whereas the coronal sequences covered only the brainstem and the basal medial sections of the hemispheres including the hippocampi.

## Statistical analyses

All statistical analyses were conducted using Statistical Product and Service Solutions (SPSS) statistics for Windows (Release 29.0; SPSS, Chicago, IL, USA). Descriptive statistics were employed to summarize data on demographic, clinical and radiologic characteristics. To assess the normality of the distribution of continuous variables, a Shapiro–Wilk test was conducted. Normally distributed data are presented as mean (SD) and compared using two sided t-tests, and non-normally distributed data are presented as median (range) and compared using the Mann–Whitney U-test and Wilcoxon signed-rank test, respectively. The significance level was set at alpha = 0.05, with a statistical trend in cases of < 0.1. Categorical variables were analyzed using the Chi-squared test and Fisher’s Exact Test, respectively.

## Results

**Fig. 1:**
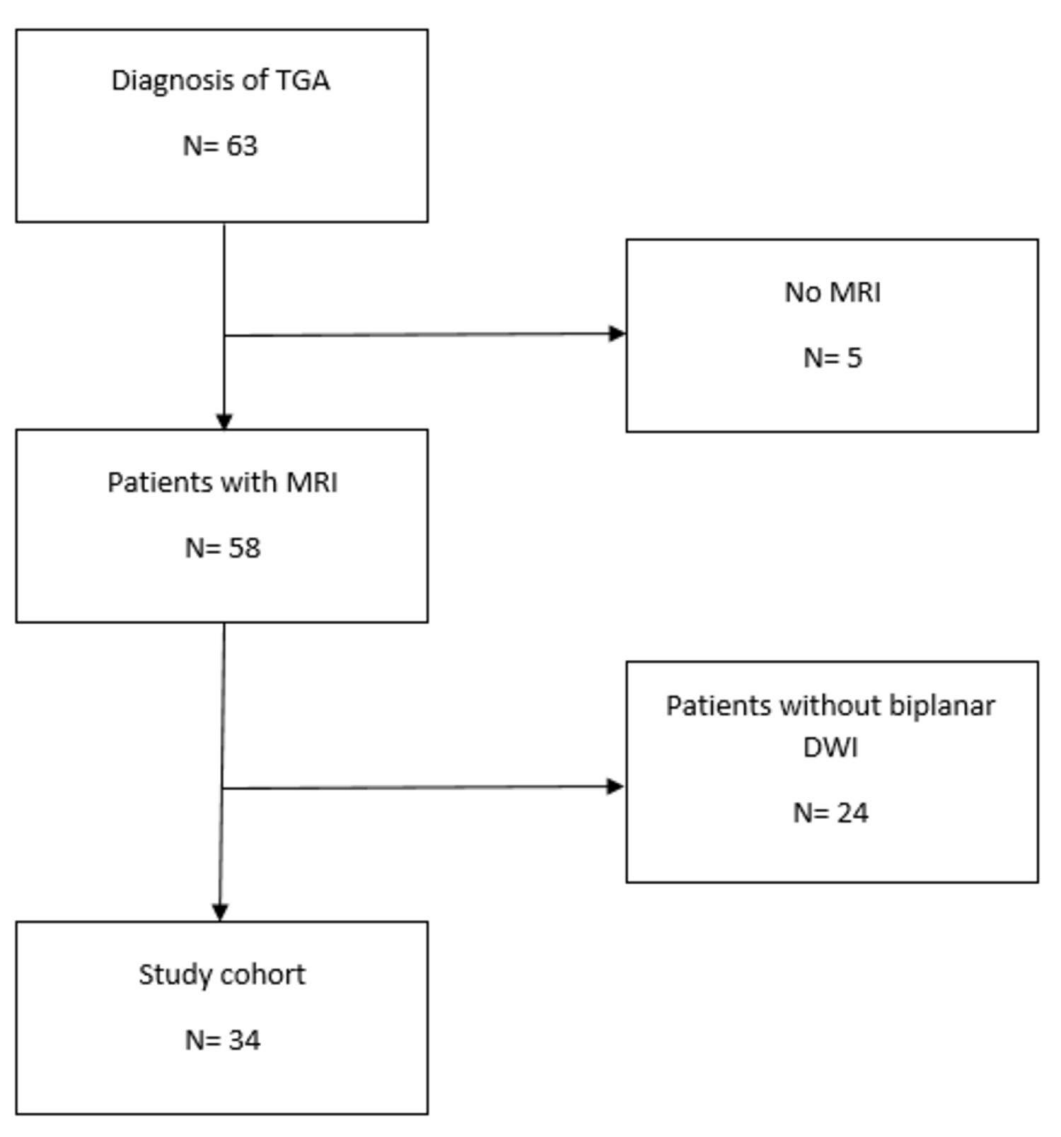
STROBE flow diagram of study participants

Overall, 63 patients were diagnosed with TGA between January 2019 and March 2024. Five patients were excluded due to the lack of MRI scans, and an additional 24 patients were excluded because biplanar MRI scans were not available. Consequently, 34 patients with TGA were available for further analyses (Fig. 1). Table 1 depicts the demographics and clinical data.

**Table 1:** The demographics and clinical data

Demographic characteristics (*n* = 34)	
Age (years) +	65.5 (8.6)
Sex	
Female*	21 (61.8)
Clinical characteristics	
Duration of Symptoms in hours°	10 (4–14)
Time until presentation in hours°	3 (2–4)
Time from onset of symptoms until MRI in hours°	34 (18–44)

+ Mean (SD)

*n (%), °median (IQR)

In the study sample, 61.8% of patients were female. The participants’ median age was 65.5 (SD 8.6) years, the median time from symptom onset to admission was 3 h (IQR 2–4 h) and the median time from onset of symptoms to MRI scan was 34 h (IQR 18–44 h) (refer to Supplementary Table 1). The median duration of symptoms was 10 h (IQR 4–14 h).

**Fig. 2:**
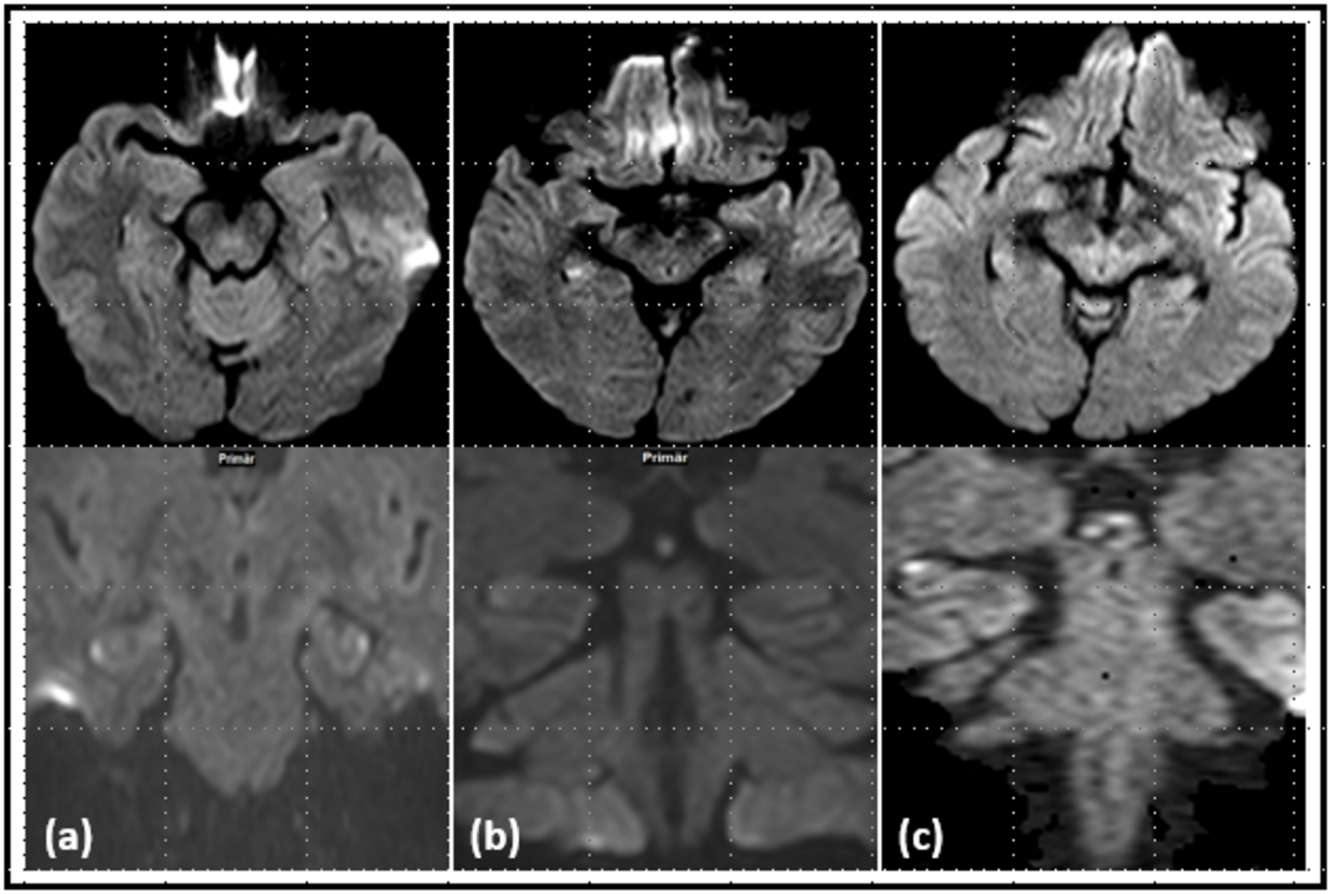
Examples of hippocampal diffusion lesion identified on axial diffusion-weighted imaging (DWI) and coronal DWI (**a**), hippocampal diffusion lesion more easily identifiable on axial weighted imaging (DWI) compared to coronal DWI (**b**) and hippocampal diffusion lesion more easily identifiable on coronal DWI (**c**)

Diffusion lesions were detected in 24 patients (70.6%). Among them, lesions were found exclusively on the axial DWI in 2 cases (5.9%), exclusively on the coronal DWI in 3 cases (8.8%), and on the coronal and axial DWI in 19 cases (55.9%) (Fig. 2). A Mann–Whitney U-Test was conducted to determine whether there were differences in time from onset symptoms to the MRI between the axial DWI lesions and non-axial DWI lesions. The distributions differed between the two groups, with a Kolmogorov–Smirnov *p* < 0.001. There was a statistically significant difference in time from symptom onset to MRI between both groups, *U* = 81,000, *Z* = − 2,146, *p* = 0.032.

Regarding the lesions’ location, 13 cases (38.2%) had left-sided lesions, 6 cases (17.6%) had right-sided lesions, and 5 cases (14.7%) had bilateral lesions (refer to Table 2).

**Table 2:** Diffusion lesions in DWI

Diffusion lesions (n = 24)	
Only present on Axial	2 (5.9)
Only present on Coronal	3 (8.8)
Axial	21 (61.8)
Coronal	22 (64.7)
Axial and coronal	19 (55.9)
Only Right-sided lesions	6 (17.6)
Only Left-sided lesions	13 (38.2)
Bilateral lesions	5 (14.7)

*n (%)

Of the 10 cases without diffusion lesions, MRI was conducted within 24 h of symptom onset in 8 cases. In 2 of the 3 cases in which the diffusion lesion was observed solely on the coronary MRI, the MRI was performed within 24 h of symptom onset. However, in the 2 cases with the diffusion lesion only detectable on axial MRI, the MRI was performed more than 48 h after symptom onset (refer to Supplementary Table 2). The side of the lesion in MRI did not correlate with the duration of symptoms, time to MRI, age or sex of the patients (Spearman’s data not shown).

## Discussion

In this retrospective study, we aimed to characterize the diagnostic yield of biplanar MRI in patients with TGA. Our findings revealed that the diagnostic yield of biplanar DWI MRI was 70.6%. In comparison, patients who underwent monoplanar MRI during the same period showed a diagnostic yield of only 61.8%, consistent with previous studies [10]. This suggests the potential significance of biplanar MRI as an essential diagnostic tool for patients with TGA.

Contrary to the previously held belief that MRI changes in TGA patients could only be detected at least 24 h after the onset of symptoms, we found that in 2 of the 3 patients who showed changes exclusively in the coronal MRI, the imaging was performed within 24 h of symptom onset [11]. This preliminary observation implies that biplanar MRI might have the potential to detect lesions earlier than previously thought. However, given the limited number of cases in our study and the need for further investigation, cautious interpretation is warranted.

Achieving a high level of diagnostic certainty through MRI in patients with other differential diagnoses, such as those with a psychiatric history, can help prevent further stigmatization. Additionally, by avoiding unnecessary anticonvulsive therapy in patients where seizure is a differential diagnosis, our study may have some clinical impact.

It is important to note that previous studies only included patients using monoplanar MRI, focusing on factors such as the strength of MRI machines (1.5-Tesla and 3-Tesla) and slice thickness, without considering the addition of coronal MRI (biplanar MRI) [10].

Our findings underscore the potential utility of biplanar MRI in diagnosing TGA. By incorporating axial and coronal imaging planes, we may enhance the detection of hippocampal lesions, providing a more comprehensive view of the brain and potentially identifying abnormalities that may be missed with monoplanar MRI. However, given the exploratory nature of our study and the limited sample size, further research with larger cohorts is warranted to validate these findings. We acknowledge that the sample size is a limitation, primarily due to the rarity of this disease and the limited geographic area served by our hospital. Our study was intended as a preliminary investigation to identify potential trends and associations within a specific cohort. Despite this, we believe the data obtained provides valuable insights and highlights the need for larger studies.

In conclusion, although our study provides initial insights into the diagnostic potential of biplanar MRI in TGA, it is essential to interpret these results with caution due to the small number of cases and the exploratory nature of the study. Nevertheless, our findings suggest that biplanar MRI may offer promise as a valuable tool in the early and accurate diagnosis of TGA, emphasizing the necessity of further research to confirm and expand upon these preliminary observations.

## Supplementary Information

Below is the link to the electronic supplementary material.

Supplementary file1 (DOCX 13 KB)

Supplementary file2 (DOCX 14 KB)

## Data Availability

The datasets used and/or analyzed in the current study are available from the corresponding author on reasonable request.
