# Supplementary material for: Biplanar MRI significantly improves early detection of transient global amnesia
Source: J Neurol. 2024 Aug 31;271(10):7030–4. doi: 10.1007/s00415-024-12643-3 (PMC11447076; doi:10.1007/s00415-024-12643-3)
Supplement: Supplementary file 1 — Supplementary file1 (DOCX 13 KB) [file 415_2024_12643_MOESM1_ESM.docx]

| Patient Number | Time from onset of symptoms until MRI in hours |
| --- | --- |
| 1 | 40 |
| 2 | 23 |
| 3 | 20 |
| 4 | 62 |
| 5 | 43 |
| 6 | 93 |
| 7 | 69 |
| 8 | 17 |
| 9 | 79 |
| 10 | 20 |
| 11 | 30 |
| 12 | 43 |
| 13 | 41 |
| 14 | 46 |
| 15 | 50 |
| 16 | 17 |
| 17 | 23 |
| 18 | 56 |
| 19 | 14 |
| 20 | 18 |
| 21 | 18 |
| 22 | 41 |
| 23 | 32 |
| 24 | 45 |
| 25 | 17 |
| 26 | 19 |
| 27 | 16 |
| 28 | 14 |
| 29 | 17 |
| 30 | 38 |
| 31 | 35 |
| 32 | 43 |
| 33 | 14 |
| 34 | 43 |

Supplementary Table 1: The time from onset of symptoms and MRI in hours (for each patient.)
