# Supplementary material for: Biplanar MRI significantly improves early detection of transient global amnesia
Source: J Neurol. 2024 Aug 31;271(10):7030–4. doi: 10.1007/s00415-024-12643-3 (PMC11447076; doi:10.1007/s00415-024-12643-3)
Supplement: Supplementary file 2 — Supplementary file2 (DOCX 14 KB) [file 415_2024_12643_MOESM2_ESM.docx]

| Diffusion lesions (n=24) | <24 hours (n=18) | >24 hours (n=16) |
| --- | --- | --- |
| Diffusion lesions on Axial (n=21) | 8 | 13 |
| Diffusion lesions on Coronal (n=22) | 10 | 12 |
| Diffusion lesions only on Axial (n=2) | 0 | 2 |
| Diffusion lesions only on Coronal (n=3) | 2 | 1 |
| Diffusion lesions on both axial and coronal  (n=19) | 5 | 14 |

Supplementary table 2: Comparison of axial- coronal MRI-Finding within two timeframes: within 24 hours (<24 hours) and after 24 hours (>=24 hours).
